# Erythroblast enucleation at a glance

**DOI:** 10.1242/jcs.261673

**Published:** 2024-10-14

**Authors:** Lucas M. Newton, Velia M. Fowler, Patrick O. Humbert

**Affiliations:** ^1^ Department of Biochemistry and Chemistry, La Trobe University, Melbourne, VIC 3073, Australia; ^2^ La Trobe Institute for Molecular Science, La Trobe University, Melbourne, VIC 3073, Australia; ^3^ Department of Biological Sciences, University of Delaware, Newark, DE 19711, USA; ^4^ Department of Biochemistry and Pharmacology, University of Melbourne, Parkville, VIC 3010, Australia; ^5^ Department of Clinical Pathology, University of Melbourne, Parkville, VIC 3010, Australia

**Keywords:** Enucleation, Erythroblastic islands, Erythropoiesis

## Abstract

Erythroid enucleation, the penultimate step in mammalian erythroid terminal differentiation, is a unique cellular process by which red blood cells (erythrocytes) remove their nucleus and accompanying nuclear material. This complex, multi-stage event begins with chromatin compaction and cell cycle arrest and ends with generation of two daughter cells: a pyrenocyte, which contains the expelled nucleus, and an anucleate reticulocyte, which matures into an erythrocyte. Although enucleation has been compared to asymmetric cell division (ACD), many mechanistic hallmarks of ACD appear to be absent. Instead, enucleation appears to rely on mechanisms borrowed from cell migration, endosomal trafficking and apoptosis, as well as unique cellular interactions within the microenvironment. In this Cell Science at a Glance article and the accompanying poster, we summarise current insights into the morphological features and genetic drivers regulating the key intracellular events that culminate in erythroid enucleation and engulfment of pyrenocytes by macrophages within the bone marrow microenvironment.

## Teaser

**Summary:** We overview recent insights into the diverse cellular mechanisms underlying erythroblast enucleation, which is the unique process by which mammalian red blood cells eliminate their nuclei.

## Introduction

Red blood cells, or erythrocytes, are highly specialised cells responsible for transporting oxygen to the tissues and organs of the body. An adult human requires the generation of ∼2×10^11^ new erythrocytes per day to sustain homeostasis (Klinken, 2002; Migliaccio, 2010). Adult mammalian erythrocytes are anucleate (lacking a nucleus) biconcave disc-shaped cells – a specialisation that allows for more haemoglobin to be packaged inside the cell and provides a maximum surface area-to-volume ratio for rapid O_2_ and HCO_3_^−^ exchange (Dao et al., 2021; Hamasaki and Yamamoto, 2000; Moreau et al., 2023). Enucleation, the process by which the nucleus is removed, is a hallmark feature of erythroid terminal differentiation in mammals, with the final expulsion of the nucleus occurring within minutes (Konstantinidis et al., 2012). Together with the assembly of the specialised erythroid cortical cytoskeleton, hereafter referred to as the membrane skeleton, the expulsion of the nucleus facilitates cellular flexibility and deformability (Li and Lykotrafitis, 2014; Mohandas et al., 1980), enhancing the ability of circulating erythrocytes to survive repeated transits through the vasculature and narrow splenic endothelial slits during their ∼120 day lifespan (Dao et al., 2021; Moreau et al., 2023; Safeukui et al., 2012).

**Figure:**
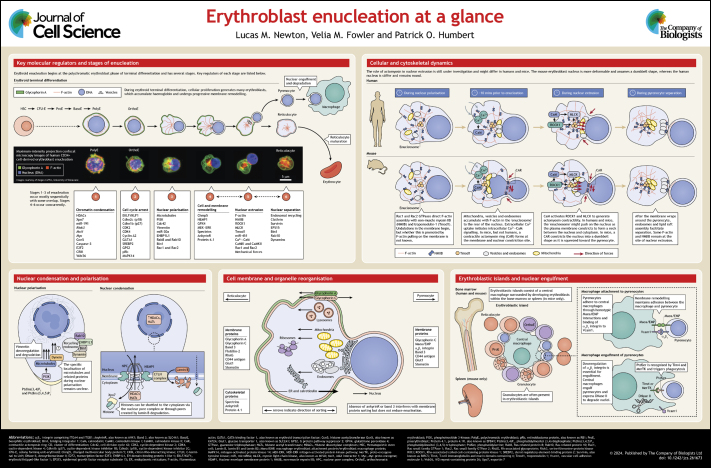
See Supplementary information for a high-resolution version of the poster.

Erythropoiesis occurs in three waves during development, each of which differs regarding the timing and mechanisms controlling enucleation (Baron et al., 2012; McGrath et al., 2008; Nandakumar et al., 2016; Palis, 2014). Here, we focus only on definitive erythroid enucleation, occurring in fetal, postnatal and adult stages, as opposed to primitive erythroid enucleation in the early embryo. We give particular attention to the events of late erythroid differentiation following the induction of chromatin condensation and cell cycle exit (late-stage erythroblast to reticulocyte). Studying these events is not without challenges. Factors including the small size of fully differentiated adult erythrocytes as well as progressive changes in transcription, cell cycle activity and growth control during terminal differentiation result in a heterogeneous and developmentally asynchronous cell population *in vivo* and *in vitro* (Godard et al., 2024; Huang et al., 2020; Liu et al., 2013). The objective of this Cell Science at a Glance article and the accompanying poster is to overview the processes of enucleation and discuss recent insights into the morphologically distinct stages and associated key molecular regulators underpinning erythroid enucleation.

## Stages of erythroblast enucleation

Erythroblast enucleation can be broken down into sequential stages according to key molecular and morphological features. Upon entering the terminal differentiation pathway, proerythroblasts undergo four to five mitotic divisions with altered growth controls, progressively decreasing both cell and nuclear size before finally exiting the cell cycle at the orthochromatic erythroblast stage (Keerthivasan et al., 2011; Menon and Ghaffari, 2021; Migliaccio, 2010). Orthochromatic erythroblasts then undergo enucleation, which comprises a final cell division yielding a reticulocyte, the future erythrocyte containing the majority of the cytoplasm, and a pyrenocyte, containing the expelled nucleus and a thin layer of cytoplasm surrounded by a plasma membrane (see poster) (Keerthivasan et al., 2011; Simpson and Kling, 1967; Skutelsky and Danon, 1970). Enucleation is characterised by a series of rapid morphological transformations (Hebiguchi et al., 2009; Wang et al., 2012; Wölwer et al., 2016; Yoshida et al., 2005) involving nuclear polarisation and extrusion, cellular reorganisation, membrane remodelling and finally separation of the pyrenocyte from the reticulocyte (Keerthivasan et al., 2011; Menon and Ghaffari, 2021; Migliaccio, 2010).

It is important to note that because it is not currently possible to synchronise primary erythroid cultures, experimental data must be interpreted carefully to avoid erroneous conclusions about where and when specific factors might be involved in enucleation. For example, dependent on the timing of genetic manipulation, disrupting processes essential for earlier steps of terminal differentiation can indirectly impact the yield and rate or efficiency of enucleation, rather than enucleation per se. With this in mind, we will discuss our current understanding of the mechanisms involved in each of these cellular processes.

### Chromatin condensation

In preparation for enucleation, erythroblasts undergo a significant decrease in nuclear size with compaction of large regions of heterochromatin. Chromatin condensation is achieved through activity of chromatin-modifying factors and histone-modulating proteins, particularly histone deacetylase complexes (HDACs) (see poster) (Zhang and Zhong, 2014). Absence of HDACs leads to defective chromatin condensation and reduced enucleation in murine erythroblast culture models (Popova et al., 2009), and knockdown of HDAC2 and HDAC6 in mouse erythroblasts prevents nuclear condensation and enucleation (Ji et al., 2010; Li et al., 2017). HDAC5 regulates chromatin condensation in human erythroblasts, and knockdown of HDAC5 impairs enucleation and increases apoptosis of terminally differentiating erythroblasts (Wang et al., 2021). Interestingly, chemical inhibition of HDACs blocks enucleation in primary mouse erythroblasts (Wölwer et al., 2015) but enhances enucleation in the human induced pluripotent stem cell (iPSC)-derived erythroid cell line HiDEP (Soboleva et al., 2021), possibly due to regulatory differences in cell lines compared to primary cells.

Certain microRNAs (miRNAs) also regulate chromatin condensation. Overexpression of miR-191 blocks enucleation by downregulating *Riok3*, *Mxi1* and *Myc* genes, which are essential for activity of Gcn5 (also known as KAT2A), a histone acetyltransferase (HAT) implicated in chromatin condensation (Zhang et al., 2011). Condensation is partly achieved by exportin 7 (Xpo7)-dependent export of histones from the nucleus to the cytoplasm, producing nuclei almost completely devoid of protein, and Xpo7 knockdown in primary mouse erythroblasts results in larger nuclei and inefficient condensation and enucleation (Hattangadi et al., 2014). Histones also appear to exit the nucleus via caspase-3-initiated ruptures (holes) in the nuclear envelope and are then degraded in the cytosol (Zhao et al., 2016). Nuclear envelope membrane protein 1 (NEMP1) maintains nuclear envelope openings in erythroblasts that are essential for enucleation, likely allowing histone egress and chromatin condensation (Hodzic et al., 2022).

Chromatin condensation in erythroblasts is also regulated by the transcription factor E2F2, which modulates expression of citron Rho-interacting kinase (CRIK), a mitotic kinase that promotes condensation (Swartz et al., 2016). WD40 repeat-containing protein 26 (Wdr26), a core subunit of the C-terminal to LisH (CTLH) ubiquitin ligase complex (also known as the glucose-induced degradation-deficient complex, Gid), assists in degrading the nuclear envelope protein lamin B [herein referring to lamin B1 (Lmnb1) and lamin B2 (Lmnb2)] to achieve rapid export of nuclear proteins during chromatin condensation (Zhen et al., 2020). The importance of chromatin condensation for erythroblast enucleation is highlighted by observations that embryonic stem cell (ESC)-derived CD34-positive cells do not efficiently enucleate compared to their cord blood-derived CD34-positive counterparts as a result of downregulation of pathways essential for chromatin condensation (Wang et al., 2022).

### Cell cycle exit

In conjunction with nuclear condensation, cell cycle exit is essential for erythroid enucleation (see poster). Cell cycle exit in erythroblasts is predominantly attributable to activity of the transcription factor erythroid krüppel-like factor (KLF1, referred to here as EKLF/KLF1), which regulates erythroid terminal differentiation in early and late stages of erythropoiesis (Siatecka and Bieker, 2011). EKLF/KLF1 facilitates cell cycle arrest through modulation of cell cycle inhibitor proteins Cdkn2c (also known as p18) and Cdkn1b (also known as p27) (Gnanapragasam and Bieker, 2017; Gnanapragasam et al., 2016; Hsieh et al., 2000). Loss of the Cdkn1b interactor cyclin A2 leads to defective enucleation, with the resulting cells containing nuclear remnants termed Howell–Jolly bodies (Jayapal et al., 2016). Mitogen-activated protein kinase 14 (MAPK14, also known as p38α) also regulates enucleation through interactions with the cell cycle inhibitor protein Cdkn1a (also known as p21) and retinoblastoma protein (pRb, also known as RB1) (Schultze et al., 2011). Additionally, cyclin-dependent kinase 9 (CDK9) blocks enucleation; however, the mechanism behind this is unknown (Wölwer et al., 2015). Other factors contributing to cell cycle exit include the erythroid transcription factor GATA1, which interacts with sterol regulatory element-binding protein 2 (SREBP2) to reduce intracellular cholesterol levels, thereby inhibiting erythroid proliferation and coordinating subsequent cell cycle exit (Lu et al., 2022). However, most of these studies do not address whether failure to exit the cell cycle blocks chromatin condensation and/or nuclear polarisation, or whether cells that fail to exit the cell cycle are still able to polarise but subsequently fail to extrude their nucleus. Resolving this question will require improved methods for temporal control over stages of differentiation and live imaging of enucleating cells.

### Nuclear polarisation

Alongside chromatin condensation and cell cycle arrest, the erythroblast nucleus polarises by moving to one side of the cell (see poster). The nature of the initiating polarity signal and polarisation mechanism remains a significant open question. Although nuclear polarisation in erythroid enucleation has elicited comparisons between enucleation and asymmetric cell division (ACD), several factors critical for cell polarity in ACD are dispensable for enucleation (Box 1). As in many other cells, the erythroblast nucleus is enclosed in a cage of vimentin intermediate filaments, and downregulation of expression and increased degradation of vimentin during terminal differentiation suggests that filament disassembly enables nuclear displacement prior to enucleation (Koury et al., 1989; Ngai et al., 1984; Sangiorgi et al., 1990). Erythroid cells derived from human iPSCs and ESCs that retain vimentin are unable to enucleate (Trakarnsanga et al., 2019), similar to avian erythroid cells (Granger et al., 1982).

**Box 1. What isn't enucleation? Puzzles and questions**

Mammalian skin keratinocytes and ocular lens fibre cells both undergo denucleation, in which the nucleus undergoes autophagic degradation following activation of proteolytic and karyolitic pathways. (Rogerson et al., 2018; Wride, 2011). Although erythroid enucleation has been compared to denucleation, enucleation appears to be a unique event in which the expelled nucleus remains intact and surrounded by plasma membrane until subsequently being phagocytosed by macrophages and degraded. Despite morphological similarities between enucleation and ACD, key ACD polarity regulators, including Scribble, Par3 and Pins (Gpsm2) proteins, are not involved in enucleation (Wölwer et al., 2017). However, the apicobasal polarity protein EHBP1L1, via regulation of endosomal trafficking pathways utilising clathrin, EPS15, Rab10 and dynamins, is indispensable for enucleation (Keerthivasan et al., 2012; 2010; 2011; Wu et al., 2023).

Cell division in proliferating erythroblasts depends on microtubule-organising centre (MTOC) regulators; however, many MTOC components are dispensable for enucleation (Kobayashi et al., 2016; Traxler et al., 2018). In mice, inhibition or deletion of a conserved MTOC component, CDK5RAP2, leads to spindle defects and failure of the final erythroblast division; nevertheless, the tetraploid cells are able to enucleate, resulting in macrocytic anaemia (Tátrai and Gergely, 2022). The minus-end-directed microtubule motor dynein has also been implicated in nuclear polarisation independent of a MTOC in human erythroblasts (Kobayashi et al., 2016).

A contractile actomyosin ring (CAR), a cytoskeletal structure typically key for cytokinesis, has been reported to propel erythroblast nuclear extrusion in some but not all studies. In mouse erythroblasts, it was initially suggested that F-actin assembled into a CAR at the membrane and nuclear constriction site (Ji et al., 2008; Koury et al., 1989; Konstantinidis et al., 2012) (see poster). However, high-resolution confocal imaging has revealed more recently that F-actin foci do not form a continuous CAR in mouse erythroblasts, and it is unclear how a discontinuous CAR could provide forces for nuclear extrusion or cellular constriction (Liang et al., 2015; Nowak et al., 2017). In human erythroblasts, F-actin foci are absent from the membrane constriction site during nuclear extrusion, only appearing when the pyrenocyte is separated from the reticulocyte (Liang et al., 2015; Nowak et al., 2017; Trakarnsanga et al., 2019). Furthermore, although mDia2 localises to cleavage furrows in dividing mouse erythroblasts, it does not localise with F-actin at the putative CAR or to the F-actin-rich enucleosome (Nowak et al., 2017). Thus, nuclear extrusion is unlikely to involve a typical cleavage furrow.

Future studies using super-resolution and/or expansion microscopy (Chen et al., 2015; Hümpfer et al., 2024) as well as immunoelectron microscopy (Mayhew and Lucocq, 2008) will be required to resolve these questions. Interestingly, enucleation appears to share features with apoptosis. In particular, expression of phosphatidylserine (PtdSer) in the outer leaflet of the plasma membrane, which triggers engulfment and degradation of apoptotic cells by macrophages, also occurs on pyrenocytes as part of erythropoiesis (Yoshida et al., 2005). It is evident that mechanisms from various pathways have been repurposed to facilitate this unique cellular event.

Nuclear polarisation is proposed to depend on endosomal vesicular trafficking regulated by the apicobasal polarity regulator EHBP1L1 via interactions with Rab10, Bin1 and dynamins (Wu et al., 2023), in addition to microtubule-dependent activation of phosphoinositide 3-kinase (PI3K) at the membrane on the side of the cell opposite the nucleus, leading to local accumulation of the PI3K products phosphatidylinositol (3,4)-bisphosphate [PtdIns(3,4)P_2_] and phosphatidylinositol (3,4,5)-trisphosphate [PtdIns(3,4,5)P_3_] (Wang et al., 2012) (see poster). The effects of treatment of late-stage erythroblasts with microtubule inhibitors support a role for PI3K and microtubules in nuclear polarisation (Konstantinidis et al., 2012; Wu et al., 2023; Xie et al., 2019), although some studies have not observed a role for microtubules (Koury et al., 1989; Ubukawa et al., 2012). This could be a result of timing of drug inhibition with respect to progression of enucleation in individual cells. For example, microtubule inhibitors do not prevent enucleation of previously polarised primary mouse orthochromatic erythroblasts (Wölwer et al., 2015). Microtubule organisation and polarity are important factors for a putative microtubule-dependent mechanism of nuclear polarisation. However, imaging approaches have not revealed a consistent localisation for microtubules during nuclear polarisation, with some studies suggesting that microtubule plus ends are oriented towards the nucleus (Konstantinidis et al., 2012; Koury et al., 1989) and others suggesting that microtubule plus ends are oriented towards the plasma membrane (Wang et al., 2012). Thus, how microtubules might control nuclear polarisation remains to be elucidated (Box 1).

### Cell membrane and organelle reorganisation

Following nuclear polarisation, the orthochromatic erythroblast undergoes membrane remodelling and internal redistribution of other organelles and proteins. Organelles, including mitochondria, vesicles and endosomes, accumulate in the cytoplasm near the polarised nucleus and remain to the rear of the nucleus during extrusion (Keerthivasan et al., 2010; Koury et al., 1989; Liang et al., 2021; Simpson and Kling, 1967). As the plasma membrane gradually surrounds the protruding nucleus, specific cell surface proteins are actively sorted to the future pyrenocyte membrane to achieve subsequent uptake and degradation of the pyrenocyte by macrophages; this process has been reviewed previously (Keerthivasan et al., 2011; Moras et al., 2017; Ovchynnikova et al., 2018). Such proteins include β1 integrin (ITGB1) and the E3 ubiquitin ligase macrophage erythroblast attachment protein (Maea, referred to here as Maea/EMP), which likely promote pyrenocyte engulfment within specialised niches termed erythroblastic islands (EBIs) (Box 2; see poster) (Bell et al., 2013; Soni et al., 2006).

**Box 2. EBIs and the enucleation niche**

Erythropoiesis is thought to take place in specialised niches, termed EBIs, within erythropoietic organs (Chasis and Mohandas, 2008; de Back et al., 2014; Jacobsen et al., 2015; Li et al., 2021). EBIs consist of a central macrophage (CM) surrounded by developing erythroblasts (see poster) (Bessis, 1958; Mohandas and Prenant, 1978; Rhodes et al., 2008). More recent evidence describes a complex structure termed the ‘erythromyeloblastic island’, which additionally supports granulopoiesis (Josselsohn et al., 2023; Romano et al., 2022). In the final stage of enucleation, the pyrenocyte nucleus maintains adhesion to the CM via the membrane protein Maea/EMP, which is expressed by both the erythroblast and CM, and via α_4_β_1_ integrin, through interactions with vascular cell adhesion molecule 1 (Vcam1) on CMs (Bell et al., 2013; Sadahira et al., 1995; Seu et al., 2017; Soni et al., 2006). Conditional knockout of Maea/EMP in mouse CD169^­­^ (SIGLEC1)-positive CMs dysregulates EBI formation and enucleation, highlighting the importance of adhesion regulation by CMs (Wei et al., 2019). Shortly after enucleation, phosphatidylserine (PtdSer) appears on the outer leaflet of the pyrenocyte plasma membrane (Box 1), which is recognised by the PtdSer receptor Tim4 (also known as TIMD4) and/or the tyrosine protein kinase MerTK on CMs, triggering phagocytosis (Miyanishi et al., 2007; Toda et al., 2014; Yoshida et al., 2005). Simultaneously, pyrenocytes downregulate expression of α_4_β_1_ integrin, thereby reducing their affinity for Vcam1 and allowing engulfment (see poster) (Toda et al., 2014). The extracellular matrix is speculated to assist in maintaining proximity of the pyrenocyte and CM at this stage. Importantly, pyrenocytes contain extremely low levels of ATP, likely as a result of Ca^2+^-ATPase activity, which contributes to PtdSer externalisation and subsequent engulfment (Yoshida et al., 2005).

Macrophages in EBIs express deoxyribonuclease II (DNase II), which degrades nucleic acids in the extruded nuclei, and DNaseII-deficient mice become severely anaemic as a result of the inability of CMs to degrade ingested pyrenocytes (Kawane et al., 2001). Erythroblast enucleation can occur independently of contact with CMs and might be assisted by direct contact between erythroblasts in culture, as has been shown in cord blood-derived erythroblasts grown at high density (Choi et al., 2013). Recently, functional iPSC-derived CMs have been generated by upregulating the expression of EKLF/KLF1 (Lopez-Yrigoyen et al., 2019). Cord blood-derived CD34-positive erythroblasts, which can adhere to the iPSC-derived CMs, show enhanced enucleation in co-culture with these macrophages, and interestingly, contact between the iPSC-derived CMs and erythroblasts is not required for the increased enucleation (Lopez-Yrigoyen et al., 2019). Subsequent experiments have identified the secreted factors interleukin 33 (IL33), plasminogen activator inhibitor 2 (PAI2, also known as SERPINB2) and angiopoietin-related protein 7 (ANGPTL7), amongst others, as responsible for this effect (Lopez-Yrigoyen et al., 2019). Ultimately, EBIs play a major role in erythroid terminal differentiation and the clearance of pyrenocytes but are not strictly required for enucleation, as evidenced by the ability of erythroblasts to enucleate relatively efficiently *ex vivo*. Further investigations into EBI functionality *in vivo* should explore how other extrinsic factors and physical forces, such as shear fluid forces, might impact enucleation and pyrenocyte clearance.

Concurrently, (α1,β1)-spectrin (composed of SPTA1 and SPTB), ankyrinR (ANK1) and protein 4.1R (EPB41), the major cytoskeletal elements in erythrocytes, are assembled into the membrane skeleton of the nascent reticulocyte prior to and during enucleation; it is likely that the majority of membrane protein redistribution is associated with this cytoskeletal remodelling (Geiduschek and Singer, 1979; Ovchynnikova et al., 2018; Wickrema et al., 1994). Erythrocyte transmembrane proteins appear to be restricted to the reticulocyte via mechanisms including reticulocyte-targeted membrane insertion and co-assembly into multi-protein macrocomplexes (Satchwell et al., 2016), and/or by ubiquitous membrane insertion followed by lateral diffusion and capture by binding sites in the reticulocyte membrane skeleton (Kusumi et al., 2012; Lee et al., 2004). Accordingly, several important erythrocyte membrane proteins, particularly those known to associate with the membrane skeleton, are segregated to the reticulocyte in mouse erythroblasts, including glycophorin A (GPA), glycophorin C (GPC), the anion channel solute carrier family 4 member 1 (SLC4A1, referred to herein as band 3) and Rh-associated antigen (RhAG) (see poster) (Lee et al., 2004; Salomao et al., 2010). In contrast, Bell et al. have observed equal partitioning of band 3 and GPC, CD44 antigen, glucose transporter 1 (Glut1, also known as SLC2A1), and the human erythrocyte integral membrane protein band 7 (stomatin) between reticulocyte and pyrenocyte membranes in human erythroblasts (Bell et al., 2013). This suggests that divergent or inefficient sorting mechanisms exist for some erythrocyte transmembrane proteins and/or that sorting mechanisms may differ between mouse and human erythroblasts (Bell et al., 2013). Surprisingly, impaired membrane protein sorting associated with absence of ankyrinR or band 3 does not interfere with enucleation efficiency, indicating that enucleation can be decoupled from membrane remodelling in mouse erythroid cells (Ji and Lodish, 2012; Satchwell et al., 2016). Interestingly, the lipid raft domain-associated protein flotillin-2 is sorted specifically to the reticulocyte membrane, which could indicate an active role in redistribution of membrane proteins via lipid rafts during enucleation (Bell et al., 2013).

Of note, substantial amounts of endoplasmic reticulum (ER) and the ER-associated protein calreticulin are distributed to the pyrenocyte (Bell et al., 2013; Griffiths et al., 2012). However, most other organelles, such as mitochondria and lysosomes, are retained in the reticulocyte (see poster). These organelles, including some ER and ribosomes, are lost via degradative and exosomal trafficking pathways after enucleation (reviewed in Ovchynnikova et al., 2018; Stevens-Hernandez and Bruce, 2022). Thus, the processes involved in organelle clearance must be actively upregulated and spatiotemporally targeted to the reticulocyte prior to enucleation. For example, the mitophagy receptor Nix (also known as BNIP3L) is upregulated in differentiating erythroblasts and is responsible for mitophagy during terminal differentiation (Sandoval et al., 2008; Schweers et al., 2007), amongst other autophagy-related proteins such as the transcription factor Foxo3 and autophagy-related protein 7 (Atg7) (Liang et al., 2015; Mortensen et al., 2010; Zhang et al., 2009). Furthermore, the ubiquitin–proteasome pathway appears to play a role in enucleation and reticulocyte maturation via endosomal sorting complex required for transport (ESCRT)-III complex-mediated cytoskeletal signalling (Chen et al., 2002; Liu et al., 2021).

Because mitochondrial positioning is tightly regulated during enucleation (Koury et al., 1989; Liang et al., 2018; 2021; Simpson and Kling, 1967), mitochondrial signalling and energy dynamics likely play an important role in enucleation. Liang et al. have observed migration and perinuclear accumulation of mitochondria before and during enucleation, as previously observed using transmission electron microscopy (Nowak et al., 2017; Simpson and Kling, 1967), and have demonstrated that pyruvate-driven mitochondrial metabolism is required for enucleation (Liang et al., 2021). Goto et al. similarly have suggested that ATP production in differentiating erythroblasts depends on pyruvate dehydrogenase kinase 4 (PDK4)-mediated anaerobic glycolysis, as blocking enzymes involved in anaerobic glycolysis arrests enucleation, whereas hypoxia results in increased enucleation and higher levels of intracellular ATP (Goto et al., 2019). Perturbing mitochondrial fission 1 protein (FIS1) arrests erythroid differentiation at the orthochromatic stage, suggesting that mitochondrial dynamics are important in the lead-up to enucleation (Gonzalez-Ibanez et al., 2020). Moreover, downregulation of the outer mitochondrial membrane protein voltage-dependent anion channel-1 (VDAC1) blocks enucleation, implicating VDAC1 in erythroid differentiation (Moras et al., 2022). Thus, regulation of mitochondrial localisation supplies the energy for enucleation and ensures sufficient segregation of mitochondria to the future reticulocyte to enable its maturation.

### Nuclear extrusion

Following nuclear polarisation and membrane and organelle reorganisation, the plasma membrane wraps around the protruding nucleus as it is progressively extruded (McGrath et al., 2017). The plasma membrane of the nascent reticulocyte is highly dynamic during nuclear extrusion, undulating and folding while the nucleus moves further from the cell centre (Nowak et al., 2017; Wang et al., 2012; Wölwer et al., 2016). The plasma membrane forms a cellular constriction with a narrowing cytoplasmic neck between the nucleus (future pyrenocyte) and the nascent reticulocyte. In mouse erythroblasts, the nucleus deforms to a dumbbell shape as it progresses through the cellular constriction, whereas in human erythroblasts, the nucleus remains nearly spherical during the entire process, suggesting that human erythroblast nuclei are stiffer than mouse erythroblast nuclei (Nowak et al., 2017; Skutelsky and Danon, 1967; Wang et al., 2012). In mouse erythroblasts, immunostaining detects lamin B but not lamin A/C (encoded by *LMNA*) in the nuclear envelope; in contrast, the nuclear envelope in human erythroblasts has been found to preferentially contain lamin A/C (Nowak et al., 2017). Downregulation of lamin B with respect to lamin A/C has been reported to correlate with increased nuclear stiffness during human erythroblast maturation (Shin et al., 2013), suggesting that the presence of lamin B (but not lamin A/C) in mice could account for the ability of the nucleus to deform during enucleation.

Assembly of filamentous actin (F-actin) is essential for enucleation, based on inhibition of extrusion (but not polarisation) upon treatment with the F-actin polymerisation inhibitor cytochalasin D (Keerthivasan et al., 2010; Konstantinidis et al., 2012; Koury et al., 1989; Repasky and Eckert, 1981; Wang et al., 2012; Wölwer et al., 2015; Yoshida et al., 2005). The prominent nuclear deformation seen in mouse erythroblasts during nuclear extrusion, together with localisation of F-actin and non-muscle myosin IIB (NMIIB; heavy chain encoded by *MYH10*) at the cellular constriction site, has led to the suggestion that a contractile actin ring (CAR) propels nuclear extrusion (Ji et al., 2008; Konstantinidis et al., 2012; Koury et al., 1989). However, others have observed that F-actin and NMIIB additionally accumulate in cytoplasmic foci at the rear of the nucleus, in a structure termed the ‘enucleosome’ (Liang et al., 2015; Nowak et al., 2017). In human erythroblasts, neither F-actin nor NMIIB accumulate at the plasma membrane constriction during most of the nuclear extrusion process, appearing instead in a cytoplasmic network and bright foci at the enucleosome (see poster) (Nowak et al., 2017). These discrepancies might be due to species-specific functional differences in enucleation between mouse and human erythroblasts, as well as differences in imaging modalities, and require further investigation (Box 1).

Pharmacological inhibition of Rac1 and Rac2 GTPases reduces F-actin assembly and arrests enucleation in both mouse and human erythroblasts (Konstantinidis et al., 2012; Ubukawa et al., 2012). The formin mDia2 (also known as DIAPH3) – a known Rho and Rac GTPase effector (Lammers et al., 2008; Miki et al., 2009) – though initially implicated in F-actin-mediated enucleation, has more recently been shown to affect processes during early terminal differentiation and reticulocyte maturation but not enucleation itself (Ji et al., 2008; Liu et al., 2021). Indeed, genetic deletion of mDia2 in mice blocks erythroblast cytokinesis but not enucleation, producing binucleate cells that are able to expel both nuclei (Watanabe et al., 2013). The functionality of Rac GTPases in enucleation might depend on Aurora kinase A (AURKA)-mediated degradation of the guanine-nucleotide-exchange factor ECT2 (Xu et al., 2024); however, the downstream effectors of Rac GTPases that promote F-actin assembly during enucleation remain to be determined. The F-actin pointed-end-capping protein tropomodulin-1 (Tmod1) has been shown to colocalise with F-actin in the enucleosome, and short hairpin RNA (shRNA)-mediated knockdown of Tmod1 reduces enucleation efficiency of human erythroblasts, likely by destabilising F-actin (Nowak et al., 2017). Inhibition of the actin regulatory small Rho-related GTPase Cdc42 by the small molecule CASIN is also reported to reduce enucleation efficiency, possibly via effects on both nuclear polarisation and extrusion (Ubukawa et al., 2020).

NMIIB contractility is required for nuclear extrusion but not polarisation, based on experiments using the myosin ATPase inhibitor blebbistatin (Ubukawa et al., 2012; Wang et al., 2012; Wölwer et al., 2016). Intracellular Ca^2+^ transients are observed within ∼8–12 minutes prior to completion of nuclear extrusion, triggering downstream signalling mediated by calmodulin (CaM) (Wölwer et al., 2016). Myosin ATPase activity could be stimulated by Ca^2+^–CaM activation of myosin light chain kinase (MLCK, also known as MYLK) and subsequent phosphorylation of myosin regulatory light chain (MLC), as blocking Ca^2+^–CaM-dependent MLCK activity with the inhibitor ML7 results in reduced enucleation (Konstantinidis et al., 2012; Wölwer et al., 2016). Inhibition of Rho-associated coiled-coil-containing protein kinase 1 (ROCK1) similarly reduces phosphorylation of MLC and enucleation efficiency (Ubukawa et al., 2012).

How actomyosin contractility provides forces for nuclear extrusion is not fully resolved. Observations of live enucleating cells reveal rapid, dynamic cell shape changes (Hebiguchi et al., 2009; Wang et al., 2012). Blebbistatin treatment arrests these changes, and in cells with a partially extruded nucleus, causes the cell to round up and the nucleus to be resorbed back into the cell (Wang et al., 2012). This led Wang et al. to propose that dynamic actomyosin network contractions generate cytoplasmic pressures that push the nucleus forwards. Formation of the F-actin- and NMIIB-rich enucleosome could be a consequence of filament accumulation due to network contractions (Nowak et al., 2017). Alternatively, actomyosin assembly into a membrane-associated CAR could provide forces to deform the cell and separate the pyrenocyte from the reticulocyte, as CAR relaxation due to blebbistatin treatment would also result in cell rounding. Live imaging of enucleating erythroblasts *in vitro* and *in vivo,* along with experiments to measure forces at the membrane and in the cytoplasm and nucleus, could help shed light on these hypotheses.

### Separation of the pyrenocyte from the reticulocyte

As enucleation progresses, vesicular trafficking creates new plasma membrane that separates the pyrenocyte from the reticulocyte (Simpson and Kling, 1967; Skutelsky and Danon, 1967). Vesicles and plasma membrane invaginations accumulate to the rear of the nucleus (Keerthivasan et al., 2010; Koury et al., 1989; Liang et al., 2021; Simpson and Kling, 1967) in the same location as the enucleosome (see poster) (Nowak et al., 2017). Of note, inhibitors of vesicle trafficking block enucleation without affecting earlier steps in terminal differentiation (Keerthivasan et al., 2010). Enucleation depends on endosomal vesicle trafficking rather than ER- or Golgi-derived vesicle trafficking, as pharmacological inhibition of the former but not the latter reduces enucleation (Aoto et al., 2019; Keerthivasan et al., 2010; Wu et al., 2023). Furthermore, shRNA-mediated knockdown of clathrin or other endocytic effectors, such as EPS15, Bin1, Rab10 and dynamins, has been used to demonstrate a requirement for clathrin-coated vesicle endocytosis and endosomal pathways (Keerthivasan et al., 2010; Wu et al., 2023). Survivin (also known as BIRC5), a member of the inhibitor of apoptosis family, has been shown to cooperate with EPS15 and clathrin to direct enucleation (Keerthivasan et al., 2012). Application of super-resolution fluorescence and electron microscopy will be necessary to determine the structural relationships and dynamics of vesicles that contribute to separation of the pyrenocyte from the reticulocyte.

The MEK–ERK mitogen-activated protein kinase signalling pathway might be required for enucleation, as selective inhibition of this pathway downregulates endocytic recycling machinery and blocks enucleation in a manner that can be partially rescued by induction of vacuole formation (An et al., 2021). Additionally, lipid rafts, which are commonly associated with membrane signalling and remodelling processes, form at the site of cellular constriction in mouse erythroblasts and their assembly is regulated by Rac GTPases (Konstantinidis et al., 2012). Treatment with filipin, an inhibitor of lipid raft assembly, reduces enucleation, although it is unclear whether inhibition of lipid raft assembly impacts enucleation directly (Konstantinidis et al., 2012). Recently, glutathione peroxidase 4 (GPX4), a lipid peroxidation inhibitor, has been shown to accrue at the site of cellular constriction during enucleation (Ouled-Haddou et al., 2020). GPX4 inhibition impairs enucleation and results in reduced lipid raft accumulation and MLC phosphorylation; these defects can be partially rescued by addition of cholesterol (which is concentrated in lipid rafts and required for cytokinesis), supporting a role for lipid rafts in enucleation (Ouled-Haddou et al., 2020).

## Conclusions and future perspectives

In recent years, many new developments have broadened our understanding of erythroid enucleation, revealing it to be a unique cellular phenomenon that is distinct from ACD, denucleation or apoptosis. The key stages of enucleation discussed in this Review (chromatin condensation, cell cycle exit, nuclear polarisation, extrusion, separation and pyrenocyte engulfment) do not appear to be fully sequential, but rather occur dynamically and often concurrently during enucleation. Ultimately, many questions about enucleation remain unanswered. What is the polarity signal that initiates nuclear polarisation during enucleation? How does actomyosin contraction via a CAR and/or the enucleosome provide forces to propel enucleation? How tightly linked are cytoskeletal and membrane remodelling events to cellular reorganisation prior to enucleation? What is the specific role of vesicular trafficking pathways in nuclear polarisation, extrusion and separation? Finally, where is the enucleation niche *in vivo*, and how do EBIs modulate enucleation (Box 2)? We propose that targeted approaches to understanding the molecular and cellular mechanisms, internal forces and genetic drivers involved in enucleation, as well as the relationship between enucleating erythroblasts and the bone marrow microenvironment, will be imperative in answering these questions. High-resolution live-cell and intravital imaging of enucleating erythroblasts labelled with fluorescent reporters combined with inducible genetic manipulation by CRISPR-Cas9 will help to better capture and understand this unique cellular event.

## Poster

Poster

## Panel 1. Key molecular regulators and stages of enucleation

Panel 1. Key molecular regulators and stages of enucleation

## Panel 2. Cellular and cytoskeletal dynamics

Panel 2. Cellular and cytoskeletal dynamics

## Panel 3. Nuclear condensation and polarisation

Panel 3. Nuclear condensation and polarisation

## Panel 4. Cell membrane and organelle reorganisation

Panel 4. Cell membrane and organelle reorganisation

## Panel 5. Erythroblastic islands and nuclear engulfment

Panel 5. Erythroblastic islands and nuclear engulfment
