# Supplementary figures and images for: Erythroblast enucleation at a glance
Source: J Cell Sci. 2024 Oct 14;137(19):jcs261673. doi: 10.1242/jcs.261673 (PMC11529606; doi:10.1242/jcs.261673)

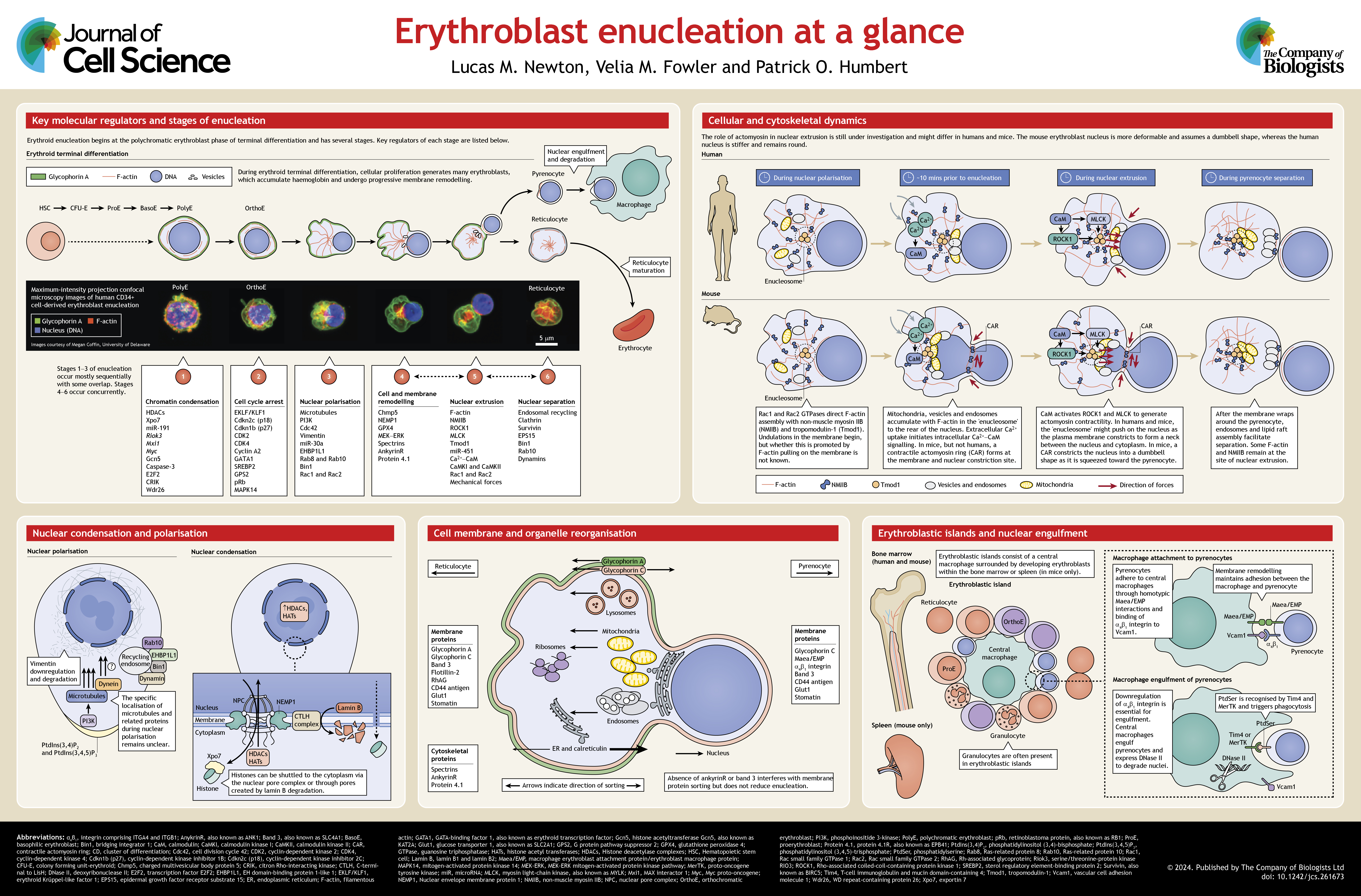

Supplement: Poster [file joces-137-261673-s1.jpg]

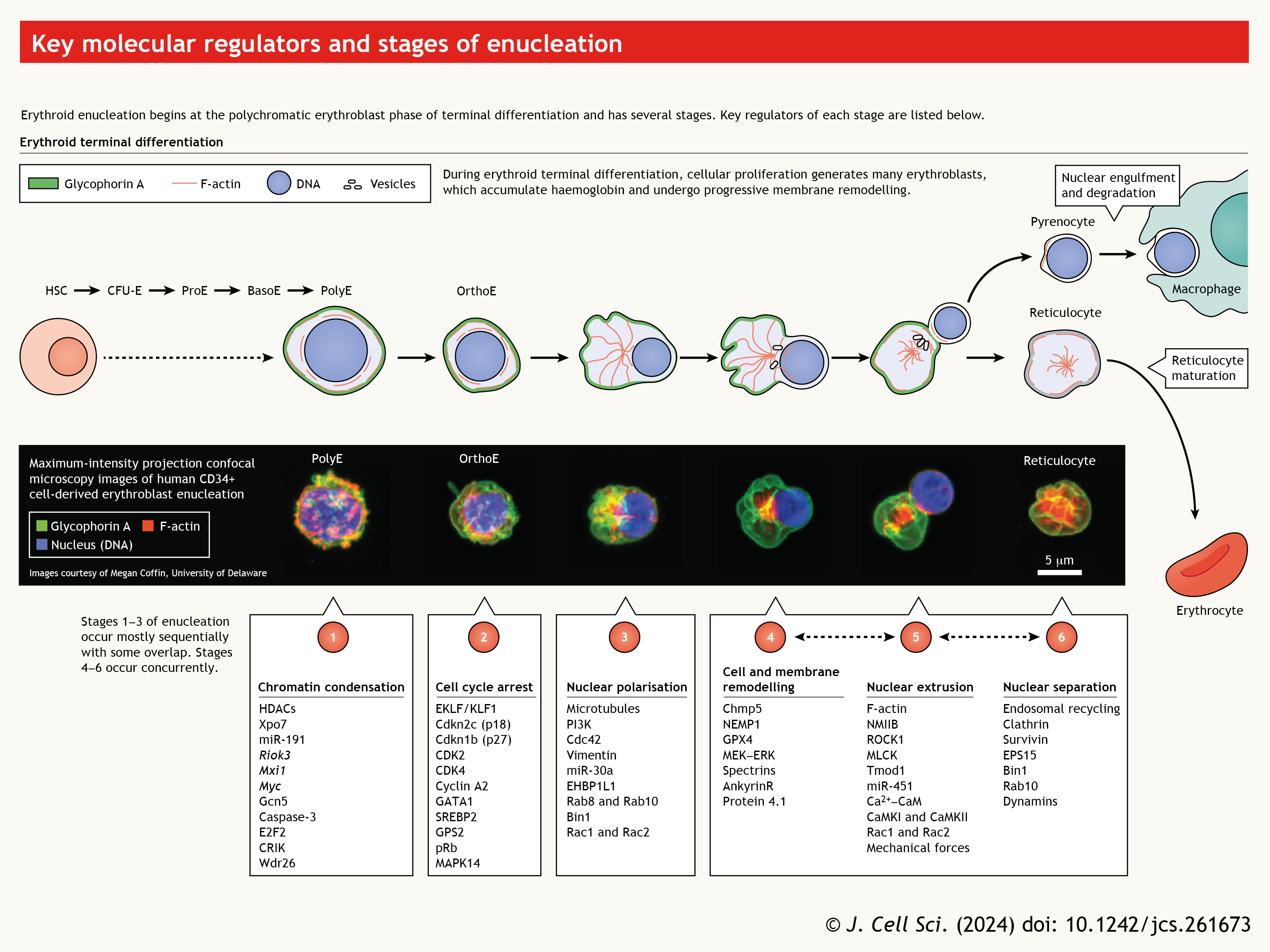

Supplement: Panel 1. Key molecular regulators and stages of enucleation [file joces-137-261673-s2.jpg]

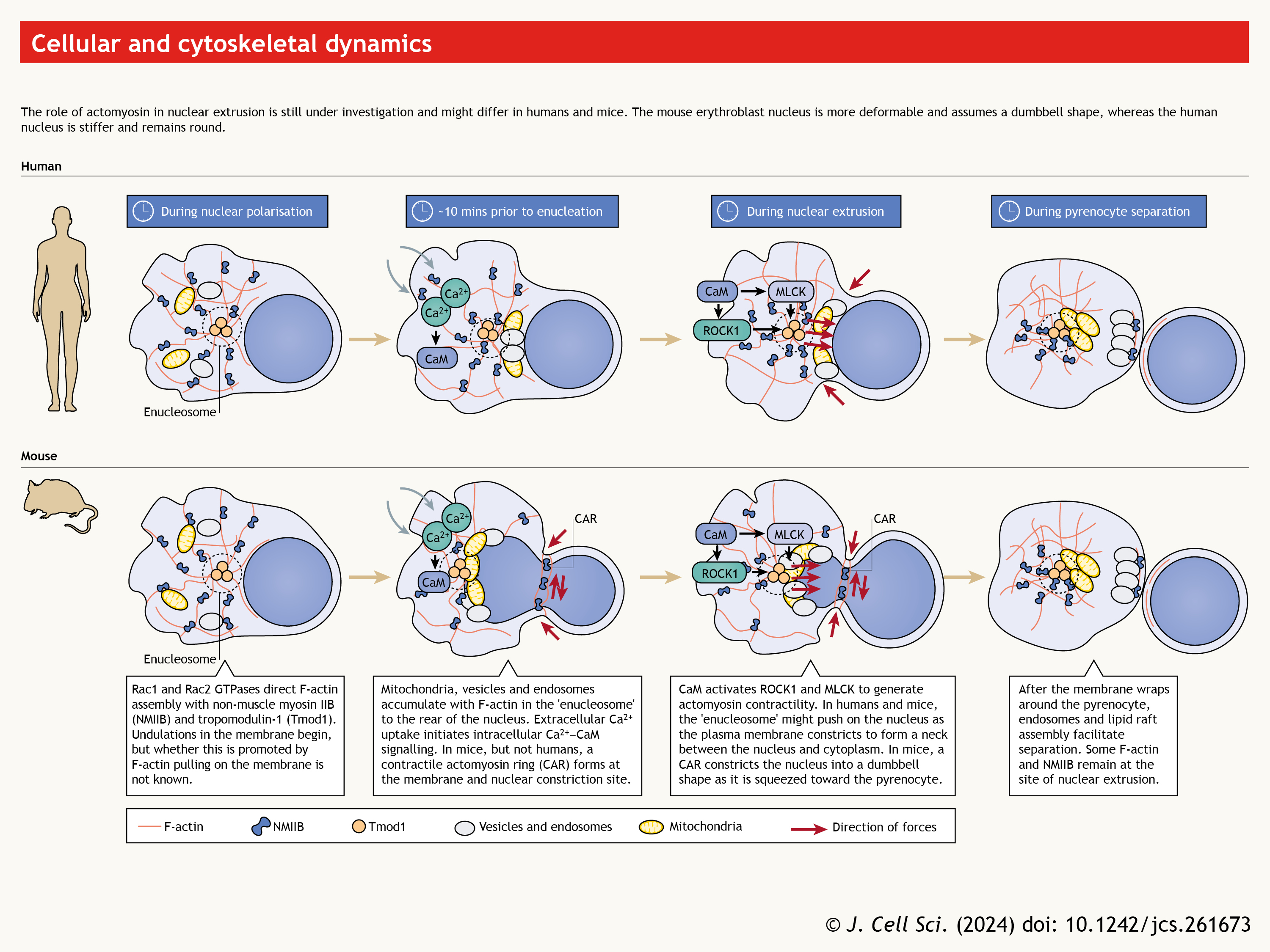

Supplement: Panel 2. Cellular and cytoskeletal dynamics [file joces-137-261673-s3.jpg]

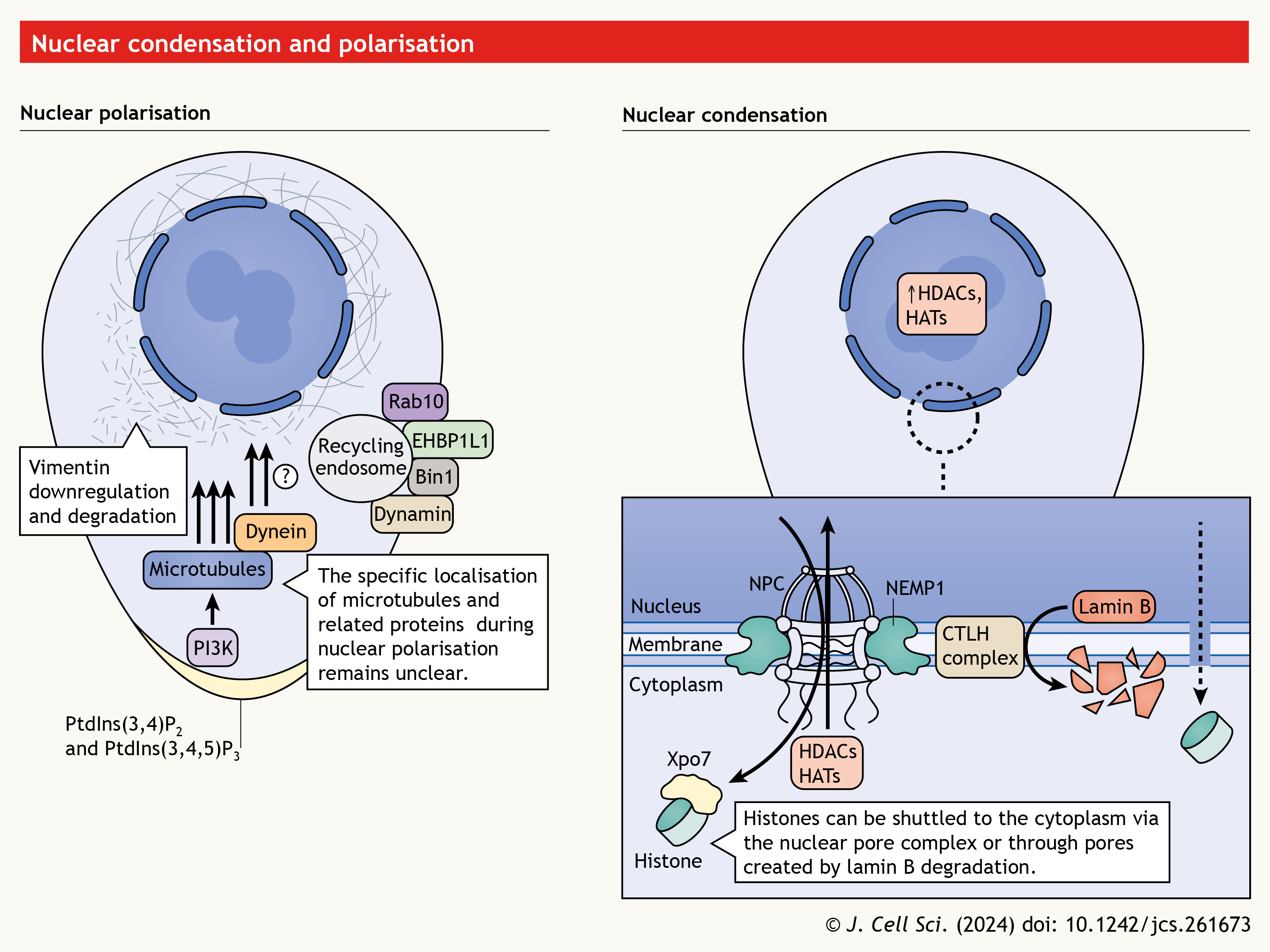

Supplement: Panel 3. Nuclear condensation and polarisation [file joces-137-261673-s4.jpg]

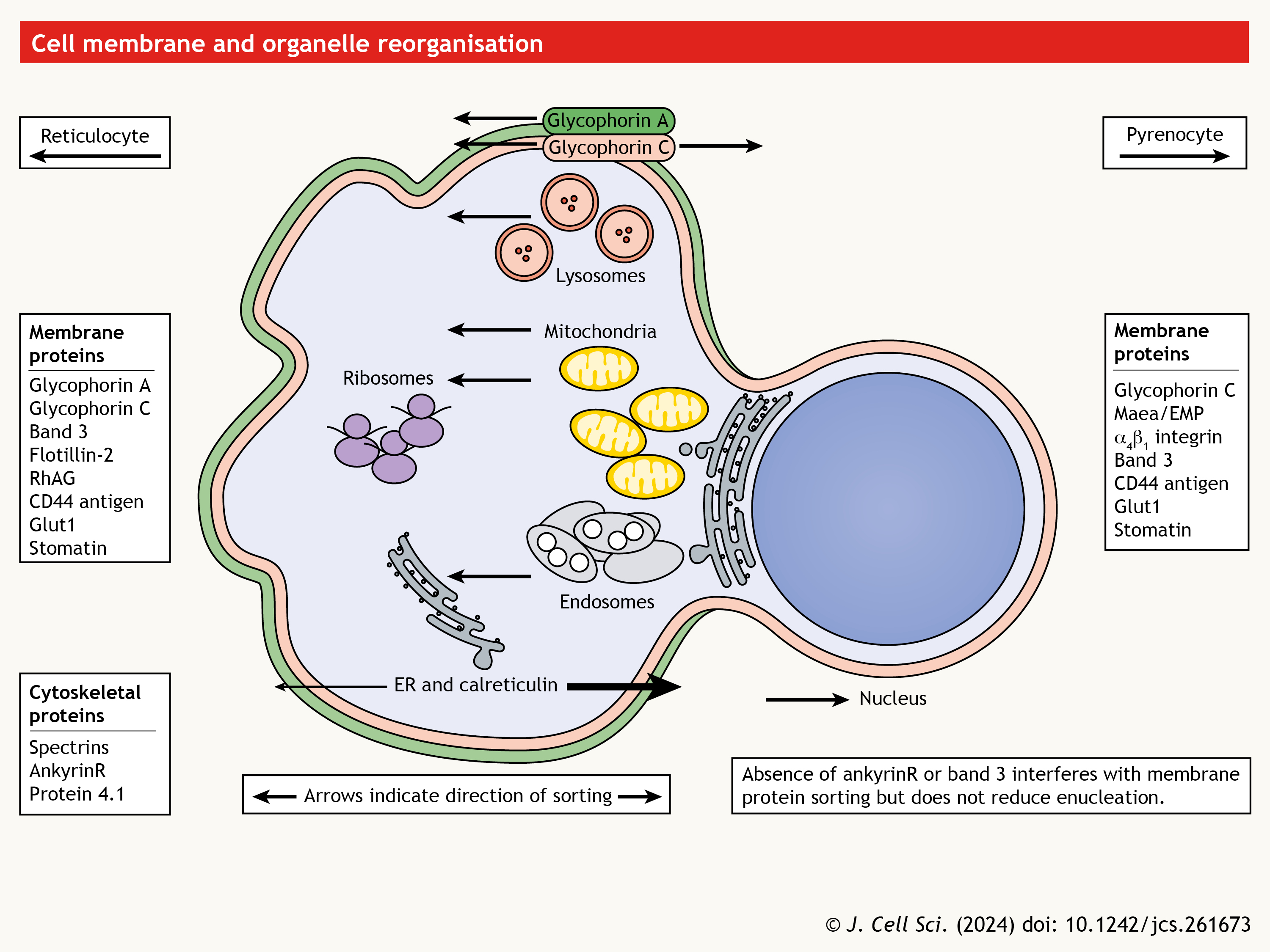

Supplement: Panel 4. Cell membrane and organelle reorganisation [file joces-137-261673-s5.jpg]

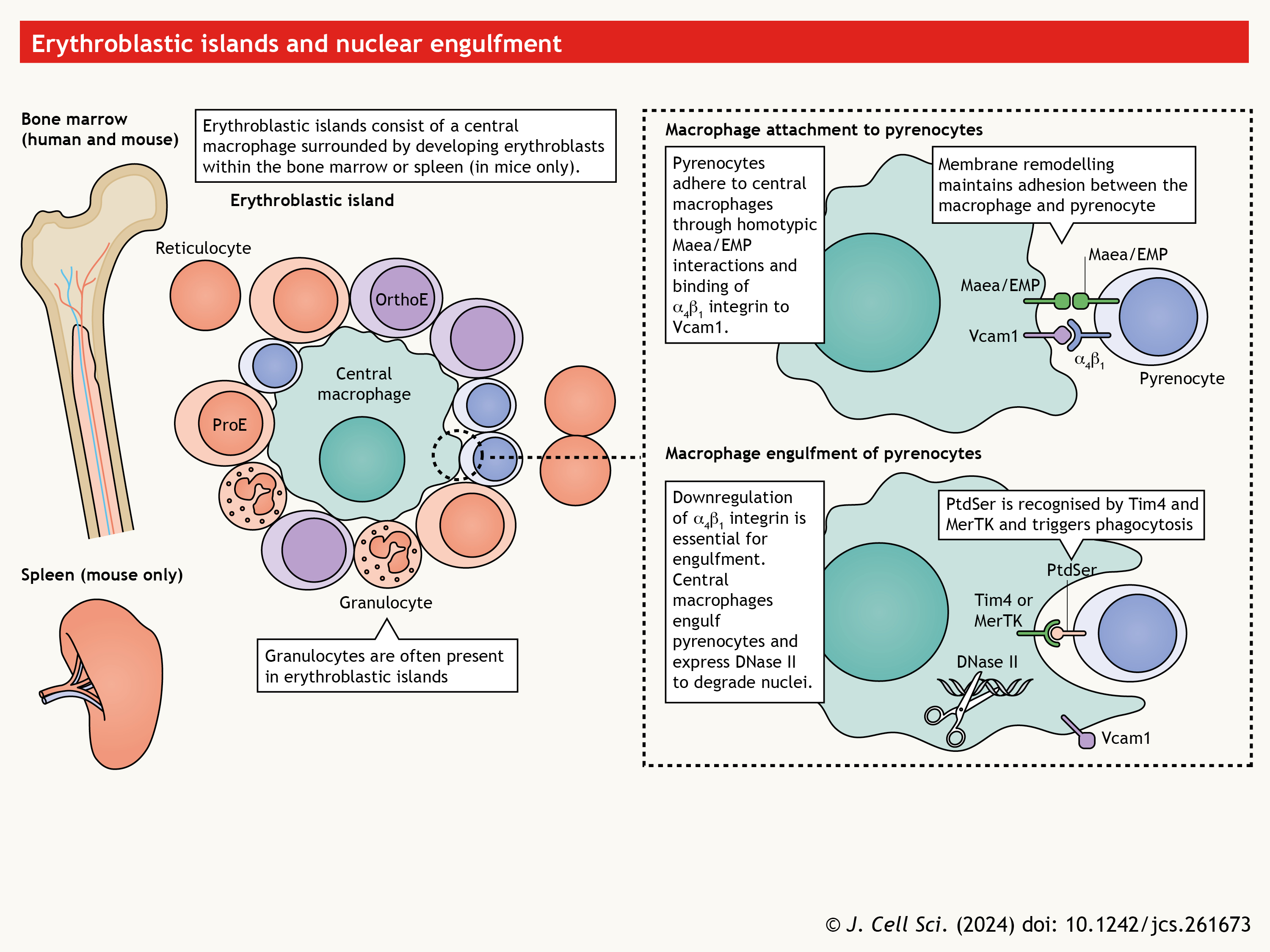

Supplement: Panel 5. Erythroblastic islands and nuclear engulfment [file joces-137-261673-s6.jpg]
